# Commentary: Technological innovations for improved prevention and diagnosis of oral disease

**DOI:** 10.3389/froh.2024.1505435

**Published:** 2025-01-20

**Authors:** Wajiha Qamar

**Affiliations:** Department of Oral Biology, Bacha Khan College of Dentistry, Mardan, Pakistan

**Keywords:** artificial intelligence, dentistry, regulatory frameworks, ethical issues, equity

**A commentary on**
Technological innovations for improved prevention and diagnosis of oral disease By Carvalho LFCS and Zanatta RF. Front Oral Health. (2024) 5:1481890. doi: 10.3389/froh.2024.1481890

## Introduction

In the editorial article titled “Technological Innovations for Improved Prevention and Diagnosis of Oral Disease”, authors Luis Felipe das Chagas e Silva de Carvalho and Rayssa Ferreira Zanatta discussed the advancements in technology that are transforming the prevention and diagnosis of oral diseases. The authors highlight the innovations including Artificial Intelligence (AI), 3-Dimensitonal (3D) printing, photonics, and salivary diagnostics which have contributed to patient outcomes, satisfaction and availability of care. Nonetheless, although the editorial provides a broad outline of such helpful technological breakthroughs, several important issues were not addressed such as regulation, equitable access, ethical issues, and the need for capacity building of health workforce. This commentary aims to provide a fair analysis of the editorial, highlighting the positive outcomes of the technologies as well as their shortcomings.

## Technological innovations in dentistry

The transformation of dental practice with the introduction of innovations such as AI and 3D printing has been effectively presented within the editorial. Artificial Intelligence (AI) holds significant promise in enhancing the diagnosis and treatment of oral diseases. Research studies have demonstrated that imaging techniques, specifically fluorescent and CT images, provide accurate analyses and can predict and diagnose diseases like oral cancer and dental plaque more effectively than traditional methods with the use of artificial intelligence (1). With these technologies, the patients have the potential of receiving accurate diagnosis, non-complicated procedures, and proper treatment plans. But still there are some challenges like low level of generalizability, data standardization and absence of external validation. It is imperative to overcome these barriers to enable the widespread uptake of AI technologies in different clinical contexts (1). For example, AI can analyze huge amounts of data helping in early detection of diseases such as oral cancer, and 3D printing helps to replace dental prosthetics and get orthodontic treatment which involves precise models (2). Also, the editorial clearly defines the importance of tele-dentistry in providing the people, especially the populations living in inaccessible areas, more so in rural areas, with oral health care. These technologies hold the potential to extend the reach of dental care to greater population segments. Nevertheless, the editorial might consider exploring some practical aspects, such as how these technologies can be diffused around the world.

## The need for regulatory frameworks

One significant issue the editorial overlooks is the necessity of having regulatory frameworks for the management of AI in dental practice. As AI technology is rapidly advancing and has been incorporated into diagnostic tools, there is a need for regulatory provisions to encourage the use of these tools in an appropriate manner (3). In the absence of such measures, there is a likelihood that patients will lose faith in AI technology due to its inability to provide consistent and correct diagnoses, or bias in AI algorithms could undermine patient trust. This rapid change in technology also creates challenges for policymakers and regulators who must adapt to such changes and ensure the safety requirements and efficacy standards (2, 3). This commentary focuses the significance of formulating and implementing suitable international strategies that will govern the utilization of AI and the innovative technologies in healthcare provision to protect the patients' wellbeing and the clinical procedures (4).

## Challenges in equitable access to technology

The challenge of equitable access of technological advancement in the field of dentistry presents another interesting topic for research. Although the editorial highly commends the promise of tele-dentistry and AI-based tools in widening the availability of services, the issue of their application in the developing world remains largely ignored. In many countries, especially rural and impoverished areas, the services necessary to facilitate this creativity such as broadband for tele-dentistry and the expensive services for 3D printers may be inconsistent. Also, the initial investment required in adopting these technologies can be too much for small dental clinics, thus increasing the disparity in low-income and high-income countries and populations (4). To ensure that such innovations do not become a privilege for a few, more work needs to be done in designing and implementing cheaper and more affordable options that can be used in many environments.

## Ethical considerations in dental technology

In the context of dental technology innovations ethical issues are equally essential, but the editorial does not explore them in sufficient detail. The rise of electronic health records and other electronic diagnostic tools raises the question of data security of patients. AI technologies depend on massive datasets to be effective; however, the problem of ensuring that patient data is properly protected while using it for AI has been a challenging task. In addition, the complexity of AI also raises concerns especially around potential bias within the systems that may affect diagnosis and treatment (5, 6). For example, an AI that uses images of human faces only from online European residents cannot be expected to perform efficiently on the African population (2, 3). The commentary highlights the need for better data management with more efficient shared responsibility vis a vis AI training using harmonious and heterogeneous data preventing an ethnicity gap.

## Educational implications for dental practitioners

Studies have found that the students who took the course on technology as part of their predoctoral classes had a better perception of digital dentistry in the end (7). This included usage of instruments including CAD, CAM systems, 3D printing and intraoral scanners which helped improve students' morale in readiness to apply these systems in their clinical settings (7). The editorial by Luis Felipe generally highlights and explains the educational aspects of technology in the workplace. However, it does not explore more about how this impacts dental practitioners. There is a need to strengthen dental education and clinical training to undertake the use of AI, 3D printing, in dental procedures. There is a growing need for dentists not only to possess clinical skills but also to master the use of complex technologies, gain competence in data interpretation, and understand ethical issues (6, 8). The commentary highlights the importance of dental schools developing their curricula in such a manner as to encompass modes of instruction that cut across fields of study. As a result, future professionals will be able to work efficiently in the face of ever-changing technological trends.

## Salivary diagnostics and microbiome studies

Recent developments have made it possible for AI, using techniques such as deep learning or machine learning, to accurately and reliably identify periodontal bone loss and assess the presence of oral cancer (1). These innovations employ frameworks like AlexNet and SqueezeNet, and report sensitivity and specificity values above 80%, thus they serve as a testament of the role AI can play in early detection and prognosis (1). Salivary diagnostics has evolved as an effective and non-invasive approach to detect various biomarkers related to a variety of conditions especially periodontal disease. According to recent research, there is a high possibility that salivary tests can be used to diagnose general inflammatory markers, which may indicate the presence of uncontrolled systemic diseases such as chronic kidney disease (7). Furthermore, microbiome research has also focused on oral and systemic relationships, as some diseases may worsen because of oral bacteria, thus helping to form a practical basis for early diagnosis and individual treatment (7). All these developments emphasize the increasing relevance of salivary diagnostics in integrative medicine.

Although the editorial's focus on salivary diagnostics and microbiome studies is rather tangential, it nevertheless holds out the promise of novel perspectives in the field of personalized dentistry. The likelihood of various cancer types and any oral illnesses can be identified far in advance of any invasive therapy by implementing such small adjustments to the diagnostic process. On the other hand, despite their advantages, salivary diagnostics technology contains many scientific and logistical problems. One major challenge is any individual's dietary intake or hydration levels will change what their salivary composition would normally be, creating additional variability in results. The diagnostic instruments are needed to undergo large scale clinical trials to evaluate their accuracy, sensitivity and specificity. The commentary reinforces that there is pertinent need to employ more advanced research that would help in perfecting these methods and help in standardizing procedures that would make it easier to rely on them in clinical practice (6).

## Multidisciplinary collaboration in dental technology

The editorial also rightly highlights that there is a crucial need to include people from other disciplines in the process of engineering technological advancements in dentistry. Different areas of knowledge such as biomedical engineering, computer science, and data science are necessary for creating and improving modern diagnostic and therapeutic methods. Nevertheless, such collaboration does not come without a cost. It is such a challenge to do composition of professionals from heterogeneous domains because of language, objectives and working culture barriers. The commentary suggests strategies to improving the collaborative culture, including the identification of common goals, channels of communication as well as mechanisms that would allow bringing resources from several disciplines under one umbrella (5, 9).

This commentary provides a more comprehensive perspective by examining the regulatory, ethical, and educational challenges posed by emerging technologies in dentistry. It also emphasizes the need to address equitable access to ensure that these innovations benefit all patients. The future of dentistry is undoubtedly bright, but careful consideration of these challenges is essential to ensure that technological advancements truly benefit all patients, regardless of their socio-economic status or geographic location.
